# Control of Directed Cell Migration after Tubular Cell Injury by Nucleotide Signaling

**DOI:** 10.3390/ijms23147870

**Published:** 2022-07-17

**Authors:** Sabrina Gessler, Clara Guthmann, Vera Schuler, Miriam Lilienkamp, Gerd Walz, Toma Antonov Yakulov

**Affiliations:** 1 Renal Division, University Freiburg Medical Center, Faculty of Medicine, University of Freiburg, Hugstetter Strasse 55, 79106 Freiburg, Germany; sabrina.gessler@gmx.net (S.G.); clara@guthmann.eu (C.G.); vera.schuler@gmx.de (V.S.); miriam.lilienkamp@freenet.de (M.L.); gerd.walz@uniklinik-freiburg.de (G.W.); 2 Signaling Research Centres BIOSS and CIBSS, University of Freiburg, Albertstrasse 19, 79104 Freiburg, Germany

**Keywords:** acute kidney injury, adenosine receptors, purinergic receptors, zebrafish pronephros model, directed cell migration

## Abstract

Acute kidney injury (AKI) is a common complication of severe human diseases, resulting in increased morbidity and mortality as well as unfavorable long-term outcomes. Although the mammalian kidney is endowed with an amazing capacity to recover from AKI, little progress has been made in recent decades to facilitate recovery from AKI. To elucidate the early repair mechanisms after AKI, we employed the zebrafish pronephros injury model. Since damaged cells release large amounts of ATP and ATP-degradation products to signal apoptosis or necrosis to neighboring cells, we examined how depletion of purinergic and adenosine receptors impacts the directed cell migration that ensues immediately after a laser-induced tubular injury. We found that depletion of the zebrafish adenosine receptors *adora1a*, *adora1b*, *adora2aa*, and *adora2ab* significantly affected the repair process. Similar results were obtained after depletion of the purinergic *p2ry2* receptor, which is highly expressed during zebrafish pronephros development. Released ATP is finally metabolized to inosine by adenosine deaminase. Depletion of zebrafish adenosine deaminases *ada* and *ada2b* interfered with the repair process; furthermore, combinations of *ada* and *ada2b*, or *ada2a* and *ada2b* displayed synergistic effects at low concentrations, supporting the involvement of inosine signaling in the repair process after a tubular injury. Our findings suggest that nucleotide-dependent signaling controls immediate migratory responses after tubular injury.

## 1. Introduction

Acute kidney injury (AKI) is a common complication of severe human disease, associated with detrimental short- and long-term consequences [1]. Despite improved supportive care, the mortality of AKI has remained largely unchanged in recent decades. The mammalian kidney encompasses an extensive capacity to recover from AKI, but attempts to facilitate the repair processes have been generally unsuccessful. Mouse models of AKI suggest that surviving renal cells, undergoing de-differentiation followed by proliferation, are mainly responsible for replacing damaged cells. Labeling studies suggest that the repair process is mainly accomplished by resident cells [2,3,4,5]. However, how surviving renal cells sense surrounding tissue damage is largely unknown since it is difficult to assess immediate repair responses in vivo due to technical limitations. In contrast, the embryonal kidney of developing zebrafish, which are transparent during the first days post fertilization, can be monitored by high-resolution time-lapse imaging in combination with fluorescent microscopy to identify early repair programs ensuing after AKI. In two-day-old zebrafish embryos, a laser-induced injury is repaired by a directed migratory program that instructs tubular epithelial cells on the anterior side of the injury to reverse their migratory pattern until a connection with cells on the posterior side of the injury is made and tubular patency has been re-established [6,7].

Release of ATP and ATP metabolites represents a key signal emitted by damaged cells [8]. ATP can trigger chemotaxis through activation of purinergic receptors [9]; however, ATP also serves as a source for adenosine production through consecutive phosphohydrolysis by the ecto-nucleotidases CD39 and CD73 [10].

The purinergic receptors consist of two subfamilies: purinergic P1 and P2 receptors. The purinergic P1 receptors encompass the four G protein-coupled adenosine receptors A1, A2A, A2B and A3. While A1 and A3 couple to heteromeric proteins of the Gα_i/o_ family and inhibit cAMP, A2A and A2B stimulate cAMP levels through Gα_s_ [11]. The low-affinity adenosine receptor A2B appears to be only activated in stressful conditions, when adenosine levels increase to micromolar concentrations [12]. While A1 improves renal function after ischemia–reperfusion (IR) injury, A2A receptor activation protects organs after IR injury by inhibiting inflammatory responses [11]. In contrast to the other three adenosine receptors, stimulation of A3 appears to worsen IR injury [10]. The purinergic P2 receptors encompass the ligand-gated ionotropic cation channels P2X and the G protein-coupled metabotropic P2Y family, comprising eight receptors. P2Y receptors are expressed in all segments of the nephron [13]. The P2Y receptors can be divided in two subgroups based on their coupling to Gα_q/11_ (P2Y_1,2,4,6,11_), activating the PLC β/IP_3_ pathway, or to Gα_i/o_ (P2Y_12,13,14_), inhibiting adenylyl cyclase to lower cAMP [14].

Due to degradation as well as rapid uptake by equilibrative nucleoside transporters, extracellular adenosine has a short half-life. Human tissues contain two ADA isoenzymes, ADA1 and ADA2, that convert adenosine to inosine. ADA is present in virtually all tissues; and in addition to its activity in the cytoplasm, it can associate with CD26 or adenosine receptors to function as an ecto-enzyme [15,16]. Adenosine deaminase (ADA) deficiency causes severe combined immunodeficiency (SCID), characterized by life-threatening infections from bacteria, viruses, and fungi [17].

We hypothesized that damaged tubular epithelial cells release ATP and ATP metabolites to signal damage to neighboring cells. We tested this hypothesis by systematically eliminating components of the ATP/adenosine signaling cascade by gene knockdown. We intentionally used translation (TBM)- and splice-blocking (SBM) morpholino oligonucleotides (MO) to screen putative pathway involved in damage recognition and control of directional cell migration for two reasons: first, each potential pathway is encoded by multiple family members, requiring several simultaneous gene deletions to determine the contribution of a single pathway; second, we intended to circumvent the capacity of zebrafish to compensate germline gene mutations [18,19]. Our approach revealed that overlapping components of this signaling cascade are involved in the migratory response triggered by a laser-induced tubular damage. Furthermore, we found that manipulation of purinergic signaling can potentially enhance migration-based pronephros repair.

## 2. Results

### 2.1. Adenosine Receptors Support the Repair of Laser-Induced Zebrafish Pronephros Injuries

**Figure 1:**
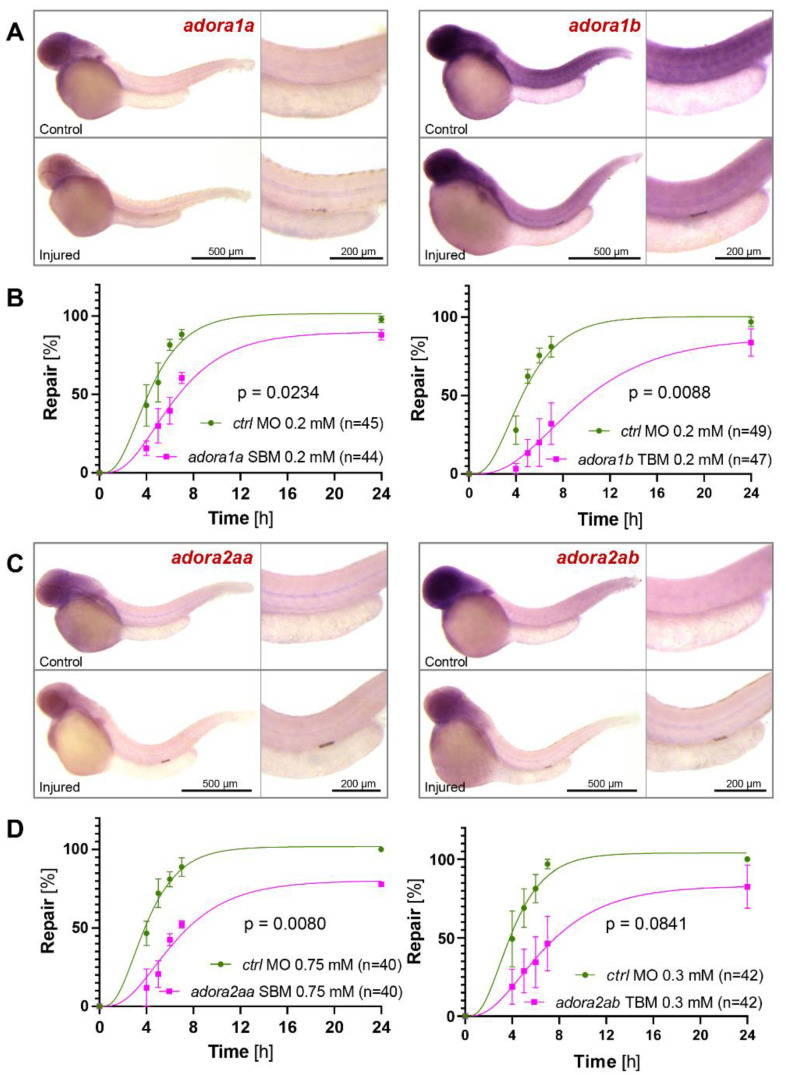
The involvement of adenosine receptors in zebrafish pronephros repair. (**A**) In situ hybridization revealed upregulation of zebrafish *adora1a* and *adora1b* after a laser-induced injury. Images were obtained two hours after the injury. (**B**) Depletion of zebrafish *adora1a* or *adora1b* by splice- (SBM) or translation- (TBM) blocking morpholino oligonucleotides (MO) delayed the repair process. (**C**) In situ hybridization revealed upregulation of zebrafish *adora2aa* and *adora2ab* after a laser-induced injury. Images were obtained two hours after the injury. (**D**) Depletion of zebrafish *adora2aa* or *adora2ab* by SBM delayed the repair process (mean ± SEM; 2-way ANOVA).

Speculating that ATP released from injured tubular cells is rapidly metabolized to adenosine by the ecto-nucleotidases CD39 and CD73, we analyzed the involvement of the zebrafish adenosine receptors in the repair process after a laser-induced tubular injury. We found increased *adora1a* and *adora1b* expression in pronephros cells adjacent to the injury suggesting a rapid upregulation of these receptors in response to injury (Figure 1A). Splice- and translation-blocking MOs to deplete these two receptors significantly delayed the repair process in 2-day-old zebrafish embryos, suggesting that both adenosine receptors are required for a normal repair response (Figure 1B). We next examined the contribution of zebrafish *adora2aa* and *adora2ab* to the repair process after laser-induced pronephros injury. Both adenosine receptors were upregulated after injury (Figure 1C). Depletion of either *adora2aa* or *adora2ab* by SBMs delayed the repair; the differences were statistically significant for *adora2aa*, but not for *adora2ab* (Figure 1D); similar non-significant results were obtained for adora2b (Figure S1A). While both A2A and A2B receptors stimulate adenylyl cyclase (AC) to generate the second messenger cAMP, adenosine A1 receptors couple to G_i/o_ and inhibit AC (Figure S1B). Despite these opposing effects on AC activity, both receptor types are required for the migration-based repair process.

### 2.2. Adenosine Degradation Represents an Essential Component of the Repair Process

**Figure 2:**
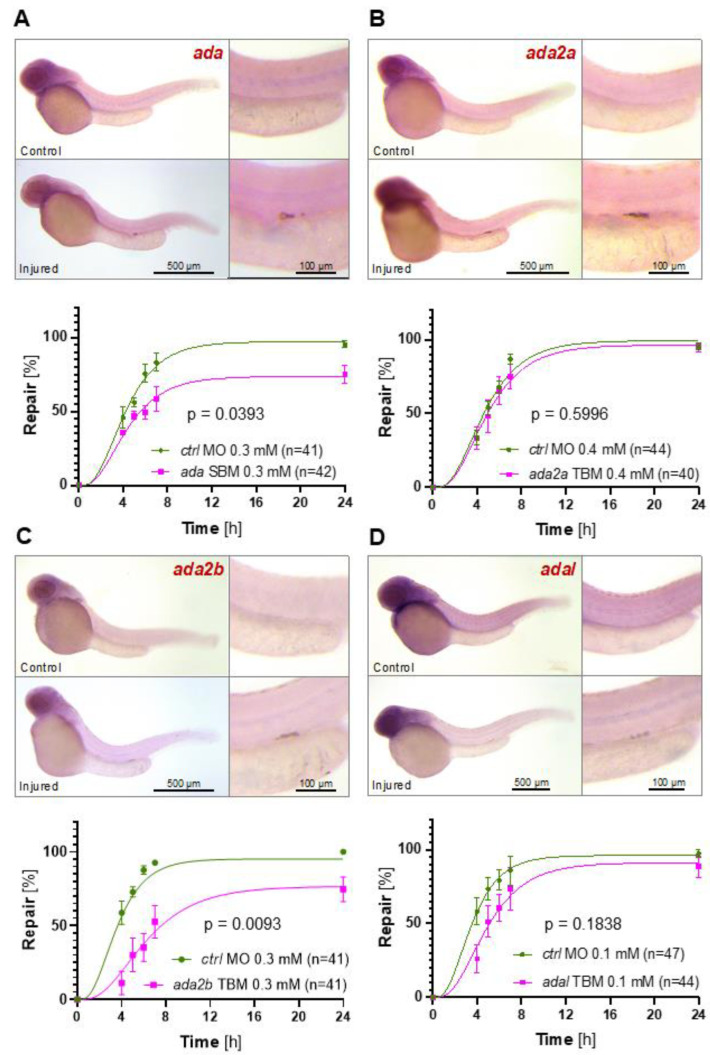
The involvement of adenosine deaminase family members in zebrafish pronephros repair. (**A**) In situ hybridization revealed upregulation of zebrafish *ada.* Depletion of zebrafish *ada* by a splice—(SBM) blocking morpholino oligonucleotides (MO) delayed the repair process. (**B**) In situ hybridization revealed upregulation of zebrafish *ada2a.* Depletion of zebrafish *ada2a* by a translation- (TBM) blocking morpholino oligonucleotides (MO) had no effect on the repair process. (**C**) In situ hybridization revealed upregulation of zebrafish *ada2b.* Depletion of zebrafish *ada2b* by a TBM delayed the repair process. (**D**) In situ hybridization revealed upregulation of zebrafish *adal.* Depletion of zebrafish *adal* by a TBM had no significant influence on the repair process (mean ± SEM; 2-way ANOVA).

**Figure 3:**
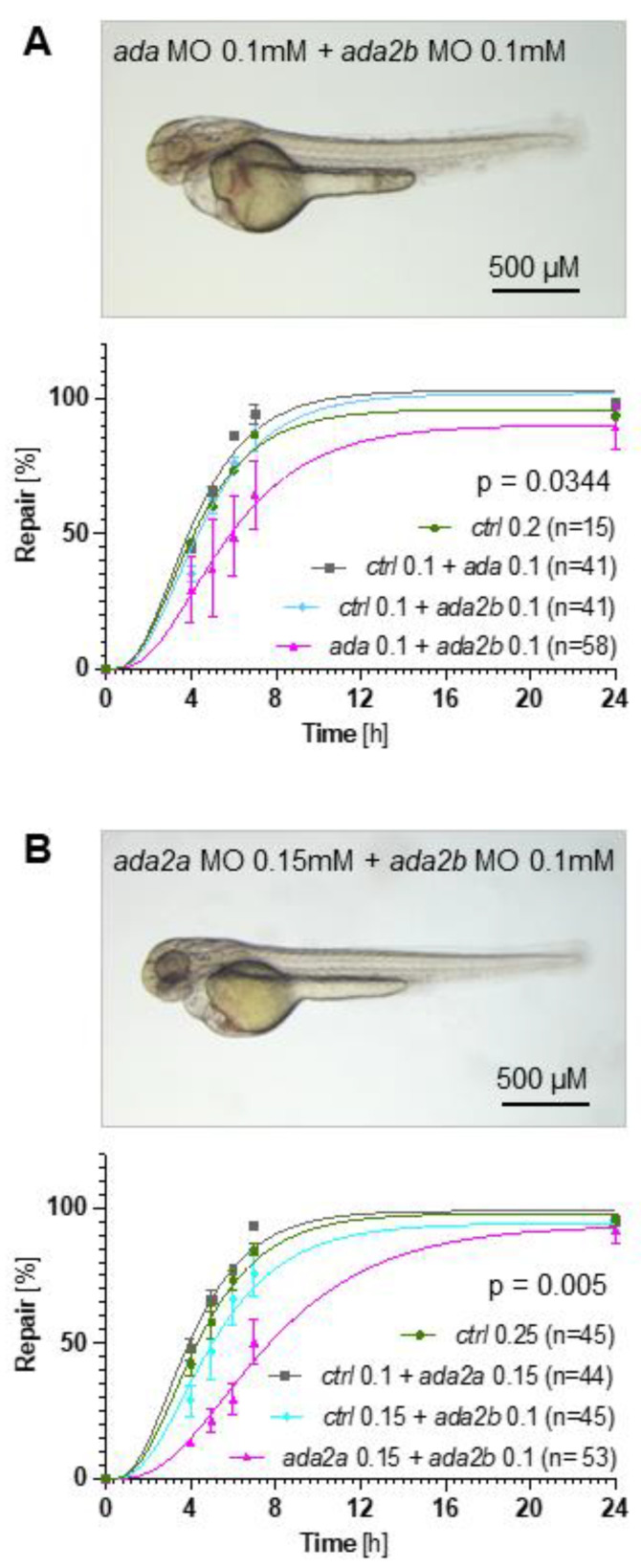
Synergistic effects between adenosine deaminase family members. (**A**) Combining low concentrations of the *ada* splice-blocking morpholino oligonucleotide (MO) and the *ada2b* translation-blocking MO did not affect zebrafish embryogenesis. However, the combination significantly delayed the repair process in comparison to the control (*ctrl*) or either single MO. (**B**) Combining low concentrations of the *ada2a* translation-blocking MO and the *ada2b* translation-blocking MO did not affect zebrafish embryogenesis. However, the combination significantly delayed the repair process in comparison to the control (*ctrl*) or either single MO (mean ± SEM; 2-way ANOVA).

Repair of a laser-induced pronephros wound critically depends on overruling the posterior-to-anterior collective cell migration that is characteristic for the developing pronephros of zebrafish embryos [20]. While cells on the posterior side of the gap increase their track speed, cells on the anterior side of the pronephros injury reverse the migratory direction, and continue to migrate in an anterior-to-posterior direction until the gap is closed and the patency of the pronephros is re-established [6,7]. Extracellular adenosine, generated from released ATP, is degraded to inosine by adenosine deaminase (ADA), affecting local adenosine concentrations [16]. Since ADA might support the formation of adenosine gradients leading to differential activation of adenosine receptors, we examined the role of ADA isoenzymes during the zebrafish pronephros repair process. Increased expression after laser-mediated injury was observed for all four zebrafish isoenzymes, *ada*, *ada2a*, *ada2b*, and *adal* (Figure 2). While depletion of zebrafish *ada* (SBM, 0.3 mM, and TBM 0.1 mM) and *ada2b* (TBM, 0.3 mM) significantly delayed the repair process (Figure 2A,C and Figure S2), depletion of zebrafish *ada2a* (TBM, 0.4 mM) and *adal* (TBM, 0.1 mM) had only a marginal effect (Figure 2B,D). Since all four isoenzymes were expressed after injury, it is likely that the depletion of one isoenzyme can be compensated by the upregulation of another family member. Combining *ada* and *ada2b* MOs at concentrations (0.1 mM) that did not affect the repair process in comparison to control embryos (*ctrl* MO, 0.2 mM), resulted in a significant repair delay (Figure 3A). Similar results were obtained for combining *ada2a* (0.15 mM) and *ada2b* MO (0.1 mM) (Figure 3B). While *ada2a* alone at MO concentrations of 0.4 mM had no effect, it significantly delayed the repair process in combination with low concentrations of *ada2b*. These results suggest that the zebrafish adenosine deaminases exert overlapping functions after pronephros injury. Furthermore, control of local adenosine concentrations by *ada* family members appears to support the repair process. To assess whether disruption of adenosine degradation affects the migratory response, we analyzed track speed and cell displacement of pronephros cells involved in the repair process by high-resolution video microscopy. In control embryos (*ctrl* MO, 0.3 mM), the laser-induced gap was almost completely repaired within 4 h, while the gap persisted in *ada2b*-exposed embryos (*ada2b*, TBM 0.3 mM) (Figure S3A). Analyses of track speed and cell displacement revealed a significant reduction in directed cell migration both in the proximal as well as in the distal segments of the pronephros (Figure S3B). Thus, adenosine signaling through adenosine receptors and degradation of adenosine by adenosine deaminases appear to be essential components of the migratory repair response in zebrafish embryos.

### 2.3. The ATP-Sensing Purinergic P2RY2 Receptor Is Required for a Normal Migratory Response after Pronephros Injury

**Figure 4:**
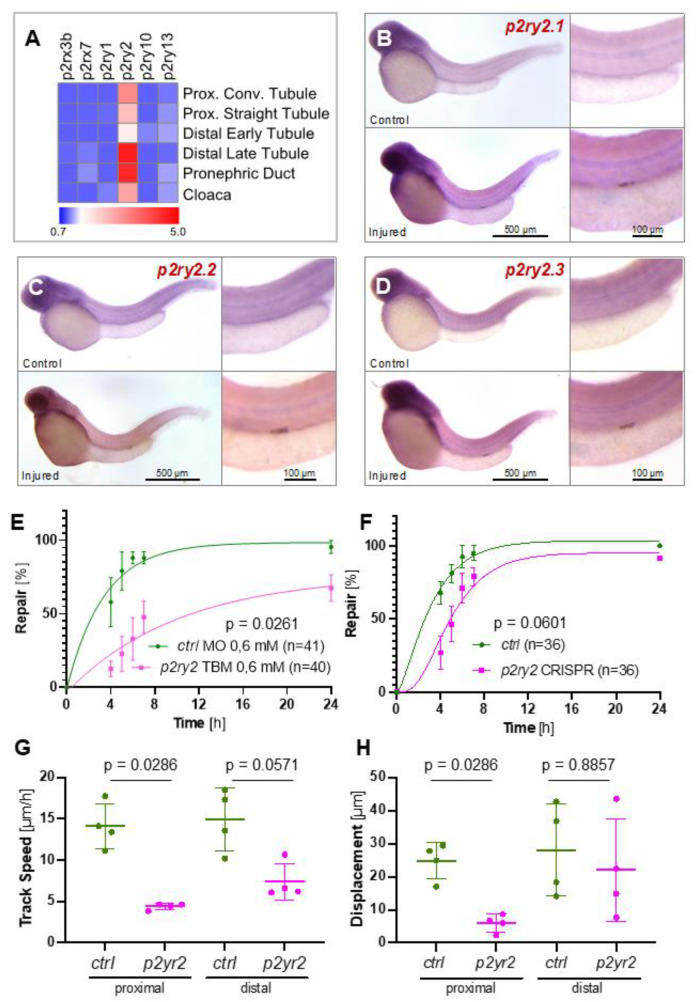
The involvement of purinergic P2ry2 receptors in zebrafish pronephros repair. (**A**) Single-cell RNA sequencing, performed with two-day-old zebrafish embryos, revealed expression of *p2ry2* along the zebrafish embryo, while other P2 family members were expressed at low levels. (**B**–**D**) In situ hybridization revealed upregulation of all three zebrafish *p2ry2* variants two hours after a laser-induced injury. (**E**) Depletion of zebrafish *p2ry2.1, p2ry2.2* and *p2ry2.3* by translation- (TBM) blocking morpholino oligonucleotides (MO) (each MO, 0.2 mM) significantly delayed the repair process. (**F**) Depletion of zebrafish *p2ry2.1, p2ry2.2* and *p2ry2.3* by CRISPR/Cas9 in combination with 8 sgRNAs delayed the repair process; however, the difference was statistically not significant (mean ± SEM; 2-way Anova). (**G**,**H**) High-resolution video microscopy revealed that *p2ry2* depletion reduced track speed and cell displacement particularly in the proximal parts of the pronephros (mean ± SD; Mann–Whitney test).

Single-cell RNA sequencing revealed expression of zebrafish *p2ry2* along the entire pronephros [21], while members of the p2x and other members of the p2y gene family were expressed at lower levels (Figure 4A). Three different genes encode for zebrafish *p2ry2* (*p2ry2*.1, *p2ry2.2*, and *p2ry2.3*). All three *p2ry2* genes were upregulated in response to injury (Figure 4B–D). The combined knockdown, using three different TBMs, resulted in a significant repair delay (Figure 4E). Similar, albeit statistically not significant results were obtained with CRISPR/Cas9-mediated gene targeting, using guide RNAs against all three *p2ry2* genes (Figure 4F and Figure S4). Time-lapse imaging revealed that a significantly reduced track speed of the neighboring tubular epithelial cells involved in the repair process was the likely cause for the defective repair process (Figure 4G,H). Notably, renal cells of embryos micro-injected with the control MO (*ctrl* MO) reassumed cellular contacts within 6 h after laser ablation, while the laser-induced gap between the anterior and posterior end of the injured pronephros remained clearly detectable (Figure S5, Movies S1 and S2).

### 2.4. The Adenosine Pathway Is Required for the Pronephros Repair Process

**Figure 5:**
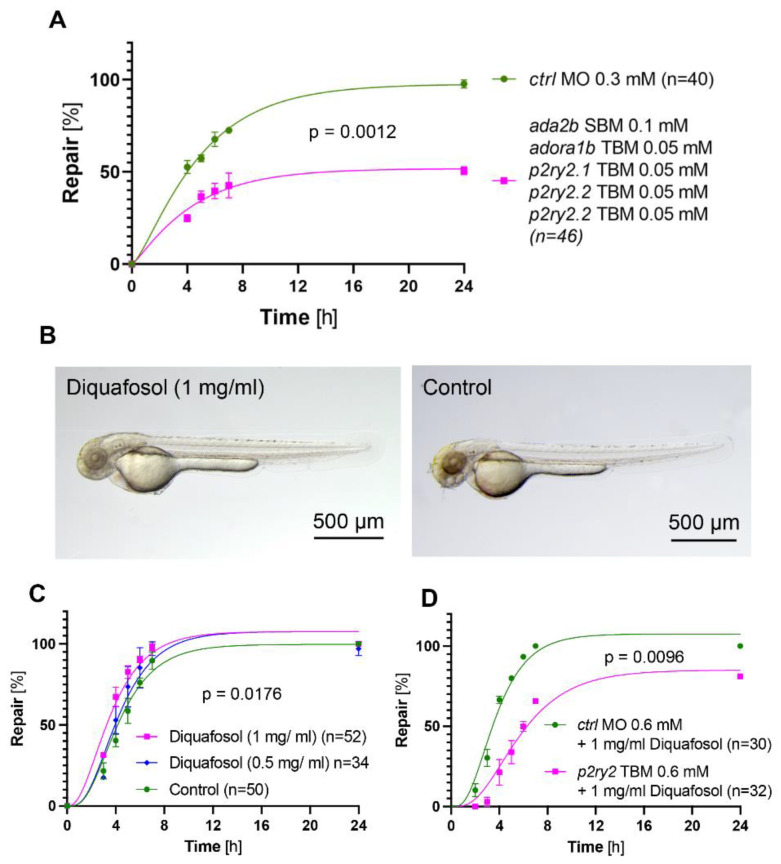
Effect of the combined adenosine pathway knockdown, and of the P2YR2 agonist Diquafosol on the repair process. (**A**) Combined depletion of *ada2b*, *adora1b*, *p2ry2.1*, *p2ry2.2* and *p2ry2.3* with low concentrations of MOs significantly delayed the repair process (mean ± SEM; 2-way Anova). (**B**) Diquafosol (1 mg/mL) did not affect zebrafish development. (**C**) Diquafosol exposure for 3 h before, and for 24 h after laser-induced injury resulted in accelerated repair (mean ± SEM; 2-way Anova). (**D**) Depletion of zebrafish *p2ry2* variants by translation-blocking morpholino oligonucleotides (MO) prevented the accelerating effect of Diquafosol.

To highlight the importance of the adenosine pathway for the pronephros repair process, we targeted the entire pathway by combining low MO concentrations of *ada2b* SBM (0.1 mM), *adora1b* TBM (0.05 mM), *p2ry2.1* (0.05 mM), *p2ry2.2* (0.05 mM) and *p2ry2.3* (0.05 mM). The combined knockdown of components of the entire pathway had a synergistic effect and strongly suppressed the pronephros repair process (Figure 5A). Thus, the activation of the adenosine pathway is required for the normal migratory response after pronephros injury.

### 2.5. Activation of Purinergic Signaling Promotes Pronephros Repair

Diquafosol is a P2RY2 receptor agonist that has been approved for treating dry eyes disease in several countries [22]. Since P2RY2 receptor signaling appears to ameliorate ischemia–reperfusion injuries [23,24], we tested whether Diquafosol can accelerate the repair process. Diquafosol tetrasodium, dissolved in water, was non-toxic (Figure 5B). To test the effect of Diquafosol on the repair process, it was added at 0.5 and 1.0 mg/mL (1.14 mM) three hours before the injury, and continued until the end of the observation period. Diquafosol at 0.5 mg/mL only slightly facilitated the repair process, while Diquafosol at 1.0 mg/mL exerted a significant effect (Figure 5C, Movies S3 and S4). Prior depletion of the *p2ry2* (TBM, 0.6 mM) prevented the effect of Diquafosol (Figure 5D). Thus, manipulation of the P2ry2 signaling pathway can be utilized to accelerate pronephros repair processes.

## 3. Discussion

Acute kidney injury (AKI), often caused by a combination of ischemia and toxic injuries, remains an enormous medical challenge and socio-economic burden [25,26,27,28,29]. Despite progress in understanding the underlying pathophysiology, AKI continues to cause high morbidity and mortality, accounting for about 1.7 million deaths per year [30]. Although the kidney possesses an amazing capacity to recover from severe AKI, repair is often incomplete, resulting in detrimental long-term complications [1]. To improve immediate and long-term prospects, tremendous efforts have been made to characterize the mechanisms that control renal regeneration. Aiming to identify cells responsible for repairing the damaged kidney, cell labeling and genetic cell fate-tracing experiments have revealed that resident renal epithelial cells re-enter the cell cycle to replace damaged cells, involving both canonical mitosis and endoreplication [2,3,31,32,33]. Two-photon in vivo microscopy has provided additional insight into the inflammatory response ensuing after AKI [34]. However, it has not yet been possible to track individual tubular cells after kidney injury to characterize the immediate adaptive responses after cell damage and tissue necrosis.

In the embryonal zebrafish kidney, injuries are repaired by directed cell migration. While cells on the posterior side of the injury increase the speed of the ongoing posterior-to-anterior collective cell migration, cells on the anterior side reverse their direction, and only resume the posterior-to-anterior collective cell migration after the injury-induced gap has been bridged and the patency of the tubular lumen has been re-established. However, it is currently unknown how tubular epithelial cells sense damage and how the migratory response is coordinated. Single-cell RNA sequencing and microarray analysis revealed upregulation of P1 and P2 purinergic family members in the pronephros of two-day-old zebrafish embryos [7,21], suggesting that ATP and ATP metabolites are involved in the signaling events in response to injury.

We found that depletion of the high-affinity adenosine receptors *adora1a*, *adora1b*, and *adora2aa* significantly delayed the repair process. A1 and A2A/2B signal through different G proteins with opposing effects on adenylyl cyclase and cAMP production in mammalian cells; however, heteromeric interaction between adenosine receptors has been demonstrated at the structural level, resulting in important control of neurotransmitter release [35], suggesting that “biased agonism” occurs in zebrafish embryonal kidneys in response to injury, a concept to explain complex G protein-coupled receptor signaling [36]. Depletion of low-affinity zebrafish adora2b did not affect the repair process, suggesting that the adenosine signaling events likely occur within a nanomolar concentrations.

Since adenosine is rapidly removed by equilibrative nucleoside transporters or degraded to inosine by extracellular adenosine deaminases (ADA), we next depleted ADA family members. Depletion of zebrafish *ada* and *ada2b* impaired the repair after injury suggesting that a precise control of adenosine levels or formation of adenosine gradients is mandatory for a normal repair response. Since the combination of low concentrations of *ada*/*ada2b* and *ada2a*/*ada2b* MOs augmented the repair defect, adenosine deaminase family members seem to act in partially complementary and/or redundant pathways.

ATP can directly bind to P2YR2 family members. Our single-cell RNA sequencing results suggested that zebrafish *p2ry2* is expressed in the pronephros. In situ hybridization after injury revealed an upregulation of all three *p2ry2* variants (*p2ry2.1*, *p2ry2.2* and *p2ry2.3*). The combined knockdown by either MOs or CRISPR reduced the tracking speeds of pronephros cells involved in the repair response, resulting in repair delays, while stimulating P2ry2 resulted in accelerated repair. The ligand-gated ionotropic cation channel P2X4 and P2X7 appear to exacerbate ischemic AKI by triggering pro-inflammatory cytokine production and NLRP3 inflammasome activation [37,38]. Similarly, G protein-coupled metabotropic P2Y14, expressed in intercalated cells of the nephron, generate chemoattractant cytokines in response to uridine glucose, resulting in renal inflammation [39]. In contrast, P2RY2 receptor ameliorates renal fibrosis after subtotal nephrectomy [40], alleviates cerebral ischemia–reperfusion injury [24], and confers the beneficial effects of uridine-5′-triphosphate after myocardial infarction [41]. Our results support a potentially unique involvement of P2RY2 in recovery from ischemia.

## 4. Materials and Methods

### 4.1. Zebrafish Lines Maintenance and Treatment

All animal work has been conducted according to the relevant national guidelines (Regierungspräsidium, Freiburg, Germany). Zebrafish lines were maintained as previously described [42]. All studies were performed in the *Tg(wt1b:GFP)*; *Tg(cdh17:GFP)* transgenic line [7]. The *p2ry2* agonist Diquafosol (MedChemExpress, Monmouth Junction, USA) was diluted in Danieau’s buffer to the described final concentrations. Zebrafish larvae were added to the Diquafosol solution 3 h before laser ablation and were kept in Diquafosol for 24 h after ablation.

### 4.2. Data Analysis and Visualization

The RNAseq data are publicly available and the analysis has been previously described [21]. The heat map was generated in Excel. The repair timecourse graphs and the statistical analysis were performed in GraphPad Prism. The utilized statistical tests are mentioned with the respective experiment.

### 4.3. Laser Ablation, Image Acquisition and Migration Quantification

Laser-induced ablations were performed as previously described [7]. Briefly, 2-day-old zebrafish larvae were embedded in 1% low melting agarose in glass-bottom dishes. Cell ablations were performed with a 2-photon laser (Chameleon) attached to an LSM 880 Observer confocal microscope (Carl Zeiss, Jena, Germany). In total, 80 µm of the pronephros was ablated. For WISH, larvae were fixed in methanol 2 h post ablation. Confocal images were recorded with a C-Apochromat 40×/1.2 objective (Carl Zeiss, Jena, Germany). Time-lapse video microscopy was carried out at the LSM 880 microscope. Z-stacks of the injury site were recorded every 10 min. The 3D reconstruction, track speed and cell displacement were calculated in Imaris (Bitplane, Zürich, Switzerland). The tubular repair was monitored with a Leica MZ16 epifluorescent stereo microscope (Leica, Solms, Germany).

### 4.4. Whole-Mount In Situ Hybridization (WISH)

Whole-mount in situ hybridization (WISH) was performed as previously described [21]. RNA probes against *p2ry2.1*, *p2ry2.2*, *p2ry2.3*, *ada*, *ada2a*, *ada2b*, *adal*, *adora1a*, *adora1b*, *adora2b*, *adora2aa* and *adora2ab* were generated from a cDNA library from 1–2-day-old embryos. For that, gene-specific sequences were amplified by PCR and cloned into a pCRII-Topo vector (Invitrogen, Carlsbad, USA). DIG-labelled anti-sense RNAs were transcribed from linearized vectors using T3 or T7 RNA polymerases (Roche, Mannheim, Germany).

### 4.5. CRISPR/Cas9 Gene Targeting

Small guiding RNAs targeting *p2ry2.1*, *p2ry2.2* and *p2ry2.3* were designed using the online tool ChopChop (http://chopchop.cbu.uib.no, accessed on 21 January 2021). CRISPR/Cas9 gene targeting was carried out as previously described [7]. Briefly, eight sgRNAs targeting *p2ry2.1*, *p2ry2.2* and *p2ry2.3* were injected simultaneously in 1-cell-stage zebrafish embryos together with TrueCut Cas9 protein (Thermo Fisher Scientific, Vilnius, Lithuania). The efficiency of each sgRNA was evaluated by PCR followed by Sanger sequencing (not shown). Injected embryos were kept at 28 °C in Danieau’s solution and subjected to laser-induced injury at 48 hpf. The following sgRNAs were used in this study:

*p2ry2.1*_prm_gRNA1 5′-AGAGAGACTCGAATAACATG AGG-3′;

*p2ry2.1*_prm_gRNA2 5′-GTGTAGGTATGCAAACCGAG AGG-3′;

*p2ry2.1*_exon2_gRNA1 5′-ACGAGATGAAACGAGCACGA CGG-3′;

*p2ry2.1*_exon2_gRNA3 5′-CAGGGGTCGAGTGATCTTAT AGG-3′;

*p2ry2.2*_exon_gRNA1 5′-GAATCGGGGCCTGTAAGATG AGG-3′;

*p2ry2.2*_exon_gRNA3 5′-GTTGATCCTCCGCCAACTCG AGG-3′;

*p2ry2.3*_exon_gRNA1 5′-GAAACTAACCAGAGGTCGTG TGG-3′;

*p2ry2.3*_exon_gRNA2 5′-TTGCTTGATCCAGGTAGCGG AGG-3′;

### 4.6. Morpholino Oligonucleotides (MOs) Gene Targeting

MOs were designed and obtained from Gene Tools, Philomath, USA. The following MOs were used in this study:

*p2ry2.1*-TBM 5′- TCGTGATCCAGATATAGATACTTTC-3′ (this study);

*p2ry2.2-*TBM 5′- GTTGTTAAATGCTGCCATCCTGATG-3′ (this study);

*p2ry2.3-*TBM 5′-TCATTCATTTTCCTTCACTTAGTCT-3′ (this study);

*ada-*TBM 5′-TCCATTCATTTCAGCCATTGTGTTG-3′ (this study);

*ada-*SBM 5′-AACAACAGGACACCACTAACCTTAG-3′ (this study);

*ada2a-*TBM 5′-TGCATGTCTGTAAGGTAATTCAACC-3′ (this study);

*ada2b-*TBM 5′-GCTTATGCTACTCATTGCTCCCAGC-3′ [43];

*adal-*TBM 5′-AAAGAGATCCGCTTCGGTGTCCATC-3′ [44];

*adora1a-*SBM 5′-ATTAAAATCTTATTACCTCATTGGT-3′ (this study);

*adora1b-*TBM 5′-GAGAGATCCTCGGGCATTCTTGCAC-3′ [45];

*adora2b-*TBM 5′-CAATGGCGATGTAGAGCGAATCCAT-3′ [46];

*adora2aa-*SBM 5′-AGAAACACCCTTCACTCACCTAAGC-3′ [47];

*adora2ab-*TBM 5′-GTGCTATCAACCAGTGTGAAAGGAT-3′ [48];

*p53-*MO 5′-GCGCCATTGCTTTGCAAGAATTG-3 [49];

Standard control (*ctrl*) MO 5′-CCTCTTACCTCAGTTACAATTTATA-3’.

All MOs were co-injected with *p53*-MO to reduce unspecific effects [49]. A volume of 4 nl of MO diluted in 100 mM KCl, 0.1% phenol red and 10 mM HEPES (pH 7.5) was injected in zebrafish embryos at the 1-cell stage. Injected embryos were kept at 28 °C in Danieau’s solution.

## 5. Conclusions

**Figure 6:**
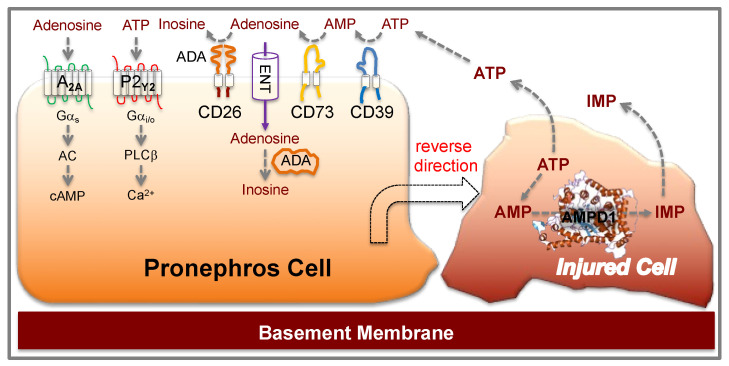
Proposed ATP-dependent signaling after a laser-induced zebrafish pronephros injury. Damaged cells release nucleotides, including ATP. Released ATP is metabolized by ectonucleoside triphosphate diphosphohydrolase-1 (ENTPD1, CD39) and ecto-5′-nucleotidase (NT5E, CD73) to adenosine. Adenosine is rapidly removed from the extracellular environment by equilibrative nucleoside transporters (ENTs), or metabolized to inosine by extracellular adenosine deaminase (ADA), associated with CD26 or adenosine receptors. Intracellular adenosine is metabolized by cytoplasmic ADA. The G protein-coupled P2RY2 receptor, signaling through Gi/o, activates PLCß, while the adenosine A2A receptor stimulated adenylyl cyclase (AC) and cAMP production through Gs. Note that other adenosine family members couple to Gi/o, inhibiting AC.

To obtain insight into the mechanism(s) that orchestrate the immediate events following a pronephros injury in zebrafish embryos, we studied the effects of ATP and ATP metabolites based on their ability to overrule the posterior-to-anterior collective cell migration that characterizes embryonal pronephros development until a laser-induced tissue gap is repaired (Figure 6). Depleting purinergic receptors delayed the repair process, revealing important roles for members of the adenosine receptor family, the ATP-degradation pathway, and for the P2 purinergic receptor P2ry2. Agonistic stimulation of the P2ry2 receptor accelerated the repair process, which might be applicable to ameliorate human acute kidney injury.

## Data Availability

The scRNAseq data were first published in [21] and are available to download from the Gene Expression Omnibus (GEO) at https://www.ncbi.nlm.nih.gov/geo/query/acc.cgi?acc=GSE162031 (accessed on 19 May 2021).

## Supplementary Materials

The following supporting information can be downloaded at: https://www.mdpi.com/article/10.3390/ijms23147870/s1.
