# Supplementary material for: Control of Directed Cell Migration after Tubular Cell Injury by Nucleotide Signaling
Source: Int J Mol Sci. 2022 Jul 17;23(14):7870. doi: 10.3390/ijms23147870 (PMC9322613; doi:10.3390/ijms23147870)
Supplement: Supplementary file 1 [file ijms-23-07870-s001.zip › Suppl. Figures.pdf]

Control of directed cell migration after tubular cell injury by nucleotide signaling

Sabrina Gessler [1+], Clara Guthmann[1+], Vera Schuler [1+], Miriam Lilienkamp [1+], Gerd Walz [1,2] and Toma Antonov Yakulov [1,*]

[1] Renal Division, University Freiburg Medical Center, Faculty of Medicine, University of Freiburg, Hugstetter Strasse 55, 79106 Freiburg, Germany; sabrina.gessler@gmx.net (S.G.), clara@guthmann.eu (C.G.), miriam.lilienkamp@freenet.de (M.L.), vera.schuler@gmx.de (V.S.), gerd.walz@uniklinik-freiburg.de (G.W.), toma.antonov.yakulov@uniklinik-freiburg.de (T.A.Y.)

[2] Signaling Research Centres BIOSS and CIBSS, University of Freiburg, Albertstrasse 19, 79104 Freiburg, Germany; gerd.walz@uniklinik-freiburg.de (G.W.)

+Equal contribution

* Correspondence: toma.antonov.yakulov@uniklinik-freiburg.de

Figure S1

A

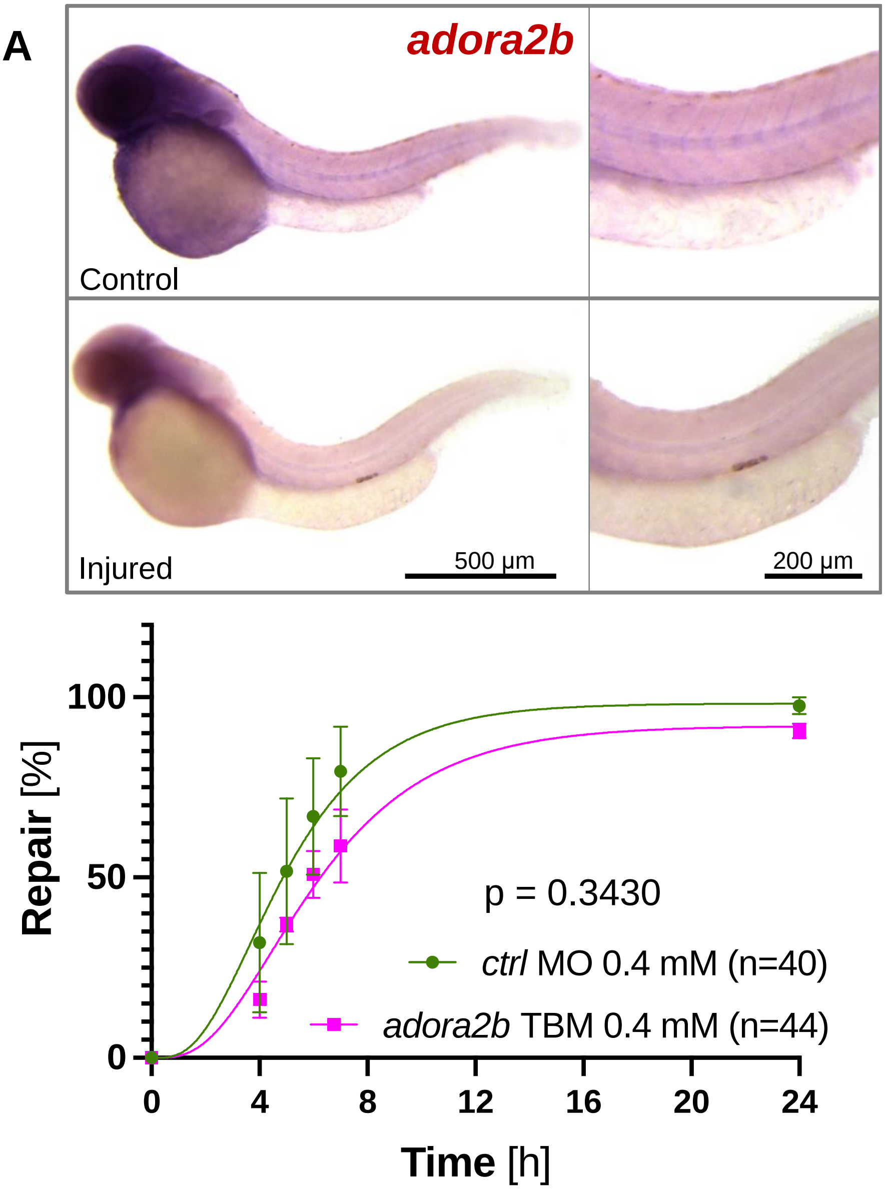
adora2b
Control
Injured
500 µm
200 µm
Repair [%]
0
50
100
Time [h]
0
4
8
12
16
20
24
p = 0.3430
ctrl MO 0.4 mM (n=40)
adora2b TBM 0.4 mM (n=44)

B

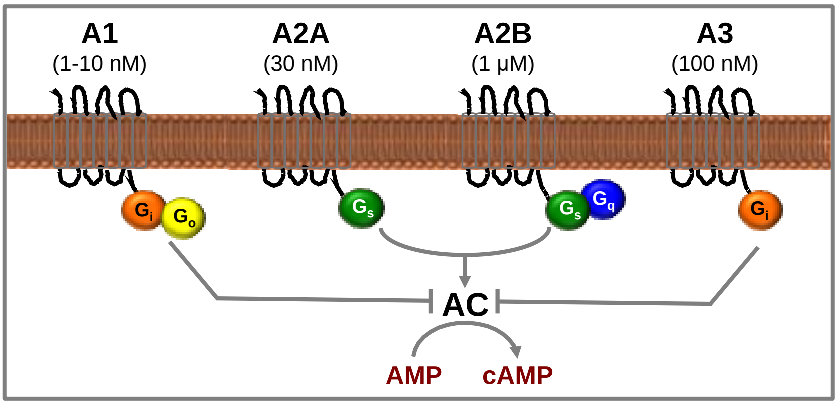
A1
(1-10 nM)
A2A
(30 nM)
A2B
(1 µM)
A3
(100 nM)
Gi
Go
Gs
Gs
Gq
Gi
AC
AMP
cAMP

Figure S2

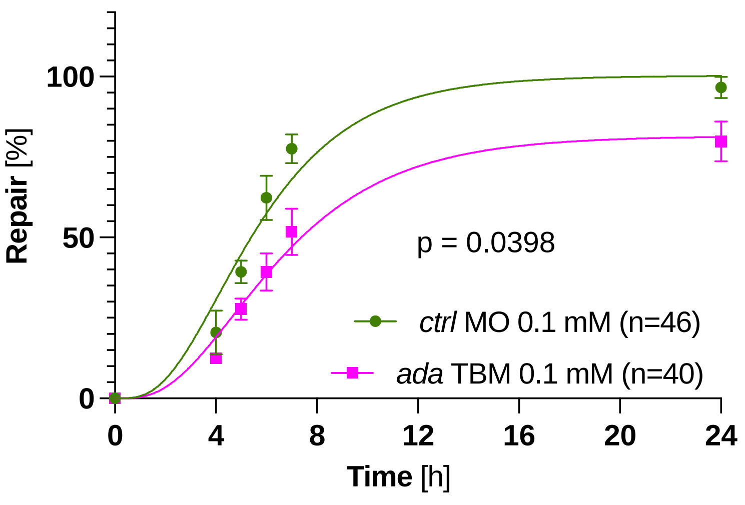

Repair [%]
100
50
0
0
4
8
12
16
20
24
Time [h]
p = 0.0398
ctrl MO 0.1 mM (n=46)
ada TBM 0.1 mM (n=40)

Figure S3

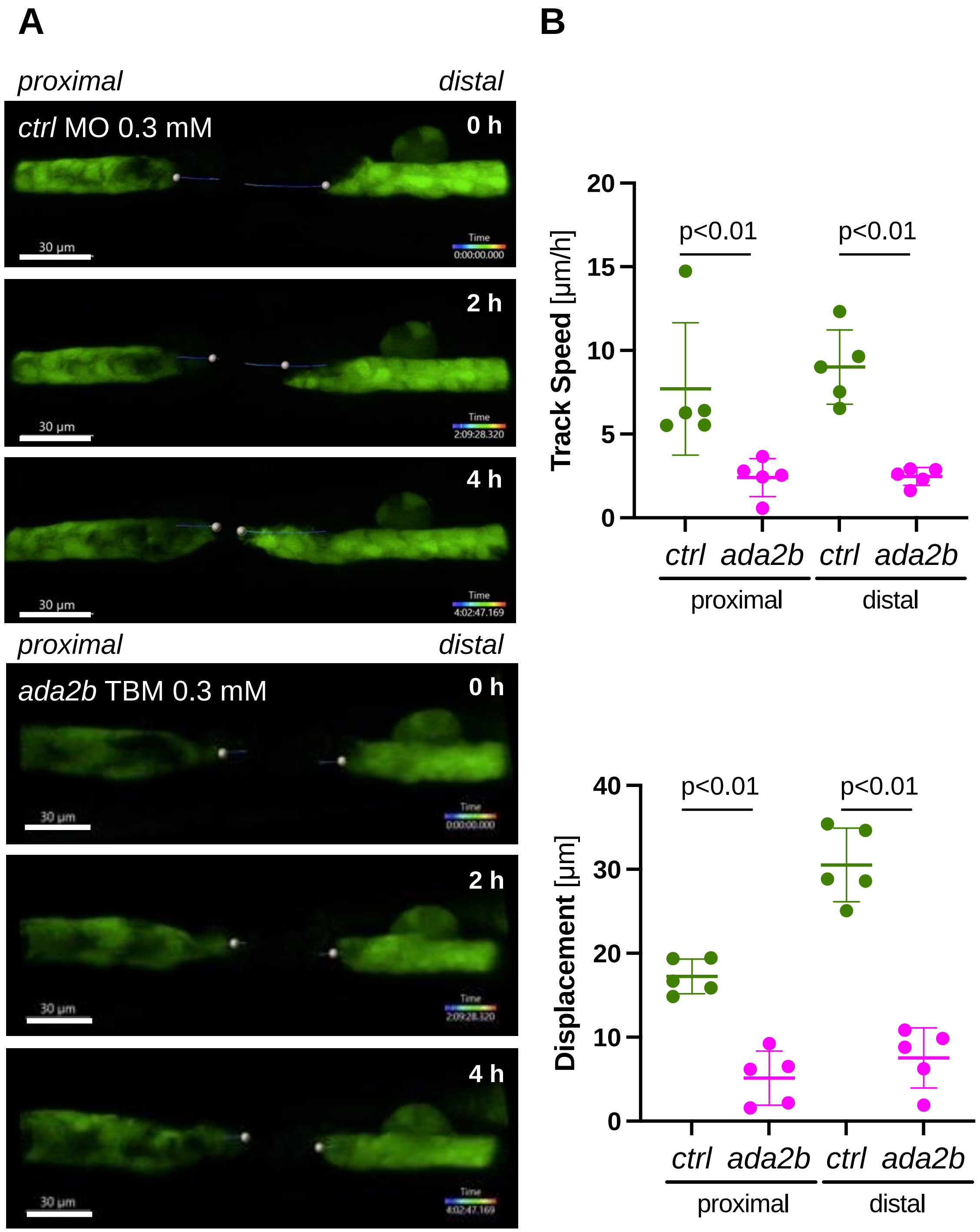
A
B
proximal
distal
ctrl MO 0.3 mM
0 h
2 h
4 h
30 µm
Time
0:00:00.000
2:09:28.320
4:02:47.169
ada2b TBM 0.3 mM
Track Speed [µm/h]
0
5
10
15
20
p<0.01
p<0.01
ctrl
ada2b
ctrl
ada2b
proximal
distal
Displacement [µm]
0
10
20
30
40
p<0.01
p<0.01
ctrl
ada2b
ctrl
ada2b
proximal
distal

Figure S4

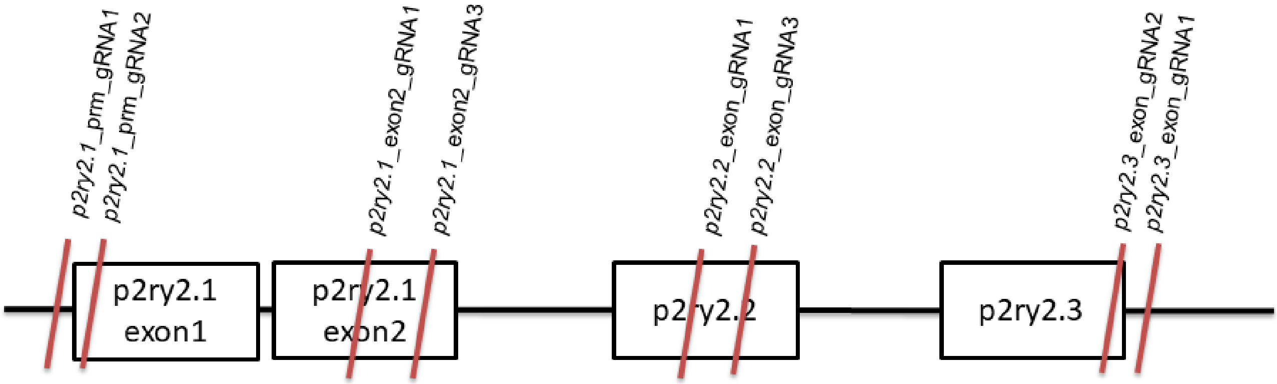
p2ry2.1_prm_gRNA1
p2ry2.1_prm_gRNA2
p2ry2.1_exon2_gRNA1
p2ry2.1_exon2_gRNA3
p2ry2.2_exon_gRNA1
p2ry2.2_exon_gRNA3
p2ry2.3_exon_gRNA2
p2ry2.3_exon_gRNA1
p2ry2.1
exon1
p2ry2.1
exon2
p2ry2.2
p2ry2.3

Figure S5

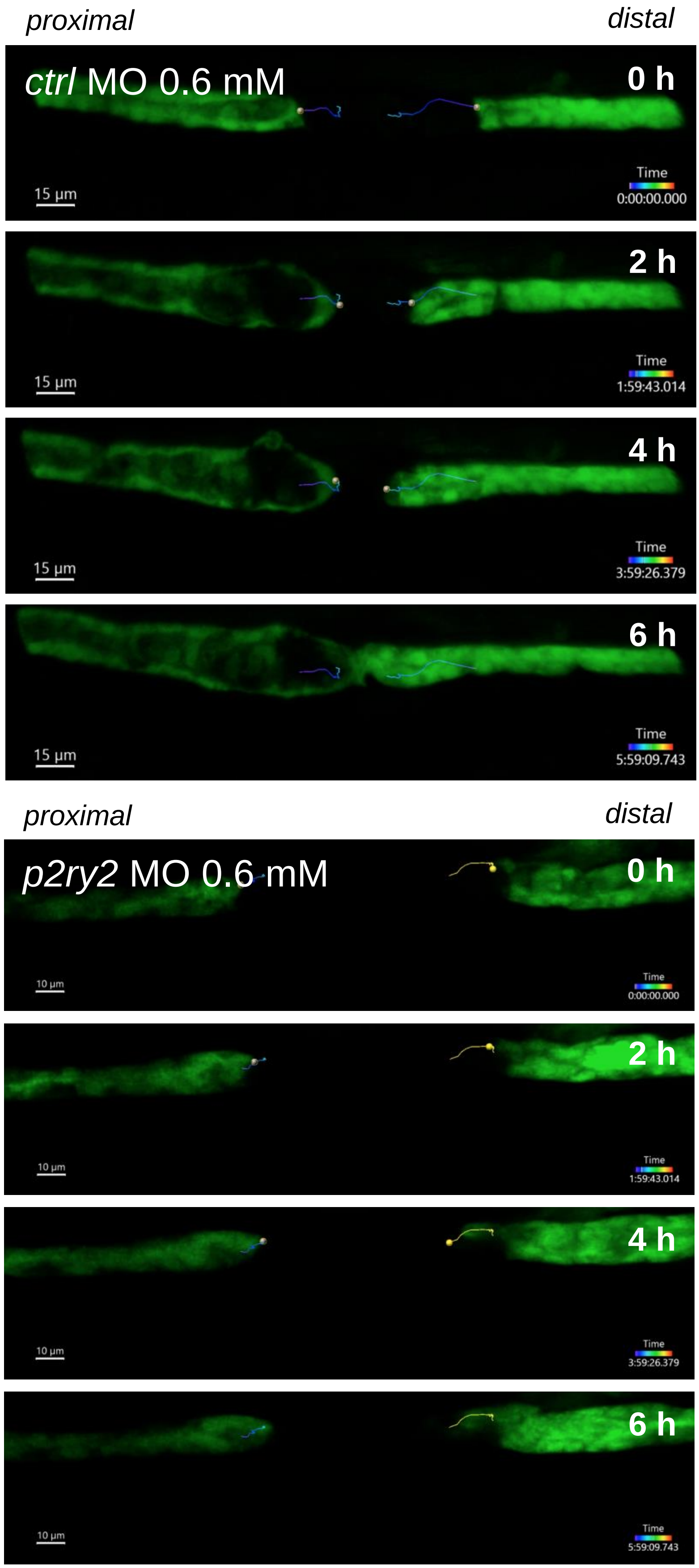
proximal
distal
ctrl MO 0.6 mM
0 h
2 h
4 h
6 h
15 µm
Time
0:00:00.000
1:59:43.014
3:59:26.379
5:59:09.743
p2ry2 MO 0.6 mM
10 µm
